# Supplementary material for: Nutrient-driven genome evolution revealed by comparative genomics of chrysomonad flagellates
Source: Commun Biol. 2021 Mar 12;4:328. doi: 10.1038/s42003-021-01781-3 (PMC7954800; doi:10.1038/s42003-021-01781-3)
Supplement: Supplementary file 2 — Supplementary Information [file 42003_2021_1781_MOESM2_ESM.pdf]

Supplement to the paper: Comparative genomics of chrysomonad flagellates reveals nutrient-driven genome evolution

Stephan Majda*, Daniela Beisser and Jens Boenigk
Department of Biodiversity, University of Duisburg-Essen, Germany

Table S1: **PacBio sequencing statistics**

species	reads [k]	bases [billions]	mean length	median length	GC%
P. encystans JBMS11	106	0.96	9040	7783	53.3
P. malhamensis	337	2.05	6086	4652	39.0
S. sphagnicola	212	1.79	8439	8031	47.6
C. danica	297	2.18	7326	7275	39.9

Table S2: **Binning and classification results**

species	assigned eukaryotic bins	assigned prokaryotic bins	MaxBin assembly size	MetaBat assembly size
C. sphaerica	7	17	132M	27M
C. fuschlensis	6	18	145M	21M
P. encystans 1006	4	30	123M	196K
P. encystans JBMS11	3	6	94M	61M
S. vulgaris	4	14	158M	41M
C. nebulosa	9	26	226M	34M
D. divergens	11	33	215M	38M
D. pediforme	6	10	144M	13M
Epipyxis sp.	4	17	101M	40M
M. annulata	13	28	171M	67M
S. sphagnicola	12	5	245M	67M

Table S3: **Comparison of the pipeline with and without additional PacBio sequencing.** Numbers reflect the amount of predicted genes belonging to different functional groups. The asterisk marks samples excluding PacBio sequences. The quotient indicates by how much the samples differ with and without PacBio. The use of long sequences increase the average contig length improving gene prediction. However, functional groups are affected to different degrees. Investigated species were *P. malhamensis* DS and *S. sphagnicola* LO234KE.

strain	DS	LO234KE	DS*	LO234KE*	DS/DS*	LO234KE/LO234KE*
Carbohydrate and lipid metabolism	962	1,392	543	1,295	1.77	1.07
Cellular processes	169	226	66	73	2.56	3.10
Energy metabolism	353	535	243	571	1.45	.94
Environmental information processing	223	1,114	116	1,651	1.92	.67
Gene set	18	73	18	95	1.00	.77
Genetic information processing	1,484	1,845	755	1,146	1.97	1.61
Metabolism	241	388	180	424	1.34	.92
Nucleotide and amino acid metabolism	1,138	1,934	731	2,147	1.56	.90
Secondary metabolism	14	60	6	71	2.33	.85

Table S4: **GC content lower in non-coding regions**

Species	Total GC [%]	Non-coding GC [%]	CDS GC [%]	GC3∗ GC [%]	Intron GC [%]	Trophy
C. sphaerica	51.3	34.4	57.2	53.5	45.9	heterotroph
C. fuschlensis	51.7	39.1	57.3	54.3	44.9	heterotroph
P. encystans 1006	54.5	39.3	58.9	55.5	45.8	heterotroph
P. encystans JBMS11	51.4	39.4	56.3	53.7	43.3	heterotroph
S. vulgaris	47.9	36.2	57	53.7	42.4	heterotroph
C. nebulosa	43.9	30.7	56.8	54.6	38.9	mixotroph
D. divergens	45.1	33.9	55.1	53.1	38.6	mixotroph
D. pediforme	51.6	37.1	58.5	54.3	44.9	mixotroph
Epipyxis sp.	34.1	27.6	50.1	48.4	32.9	mixotroph
C. danica	45.4	36.8	49.7	48.3	41.6	mixotroph
P. malhamensis	40.4	34.4	46.4	44.9	38.1	mixotroph
M. annulata	40.1	31.8	53.4	51.5	35.5	phototroph
S. sphagnicola	46.9	40.4	54.4	52.2	45	phototroph

∗ GC content based only on the third position of base triplets of gene sequences

Table S5: **Comparison of genome size estimation.** Genome size estimates based on flow cytometry and assembly differ. In general, gene density is not significant between different nutritional modes. Genome size differs significantly based on flow cytometry.

	**Genome size**		**Gene density**	
Based on method:	Genome assembly	Flow cytometry	Genome assembly	Flow cytometry
Anova_pval	0.0965	0.00133	0.556	0.0722
Tukey (M-P)	0.1995825	0.0032896	0.9987525	0.9378277
Tukey (H-M)	0.7115808	0.6908617	0.5778607	0.1085216
lm_pval	0.0344	0.000423	0.457	0.0701

Figure S1: **Additional PacBio sequencing changes proportions of gene assignment.** The strains *P. malhamensis* and *S. sphagnicola* were assembled with and without (marked by star) PacBio sequences. *P. malhamensis* grew under axenic conditions and *S. sphagnicola* did not. Functional groups were affected to varying degrees.

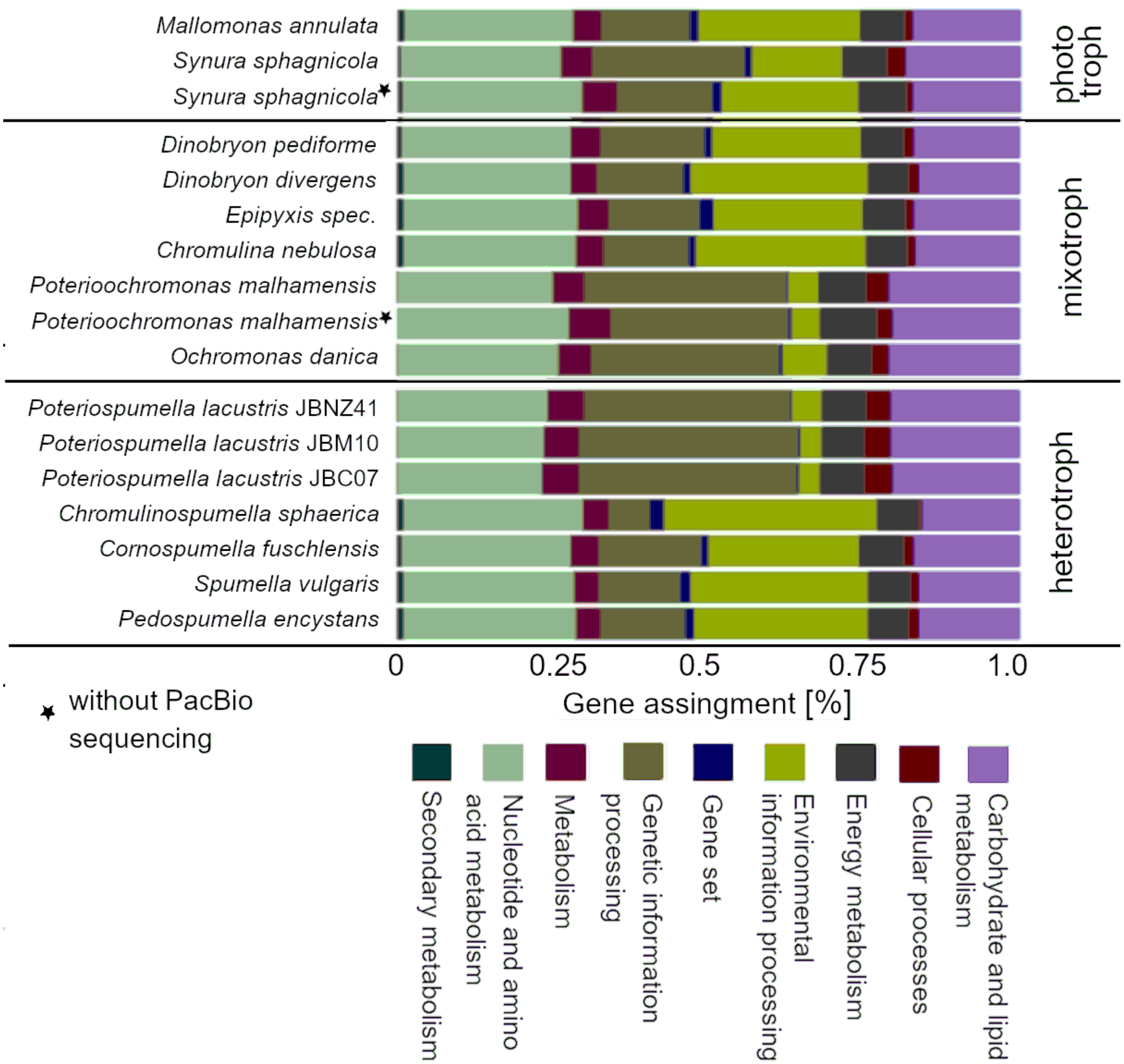

Figure S2: **Flow diagram of assembly, binning and gene prediction.** First, 16 strains were sequenced with Illumina and partly with PacBio. The reads were assembled (blue) to contigs. If the culture were non-axenic a binning step (green) filtered out prokaryotic reads. A second assembly used the filtered data. Subsequently, the genes were predicted (purple). Optional transcriptome data supported the prediction.

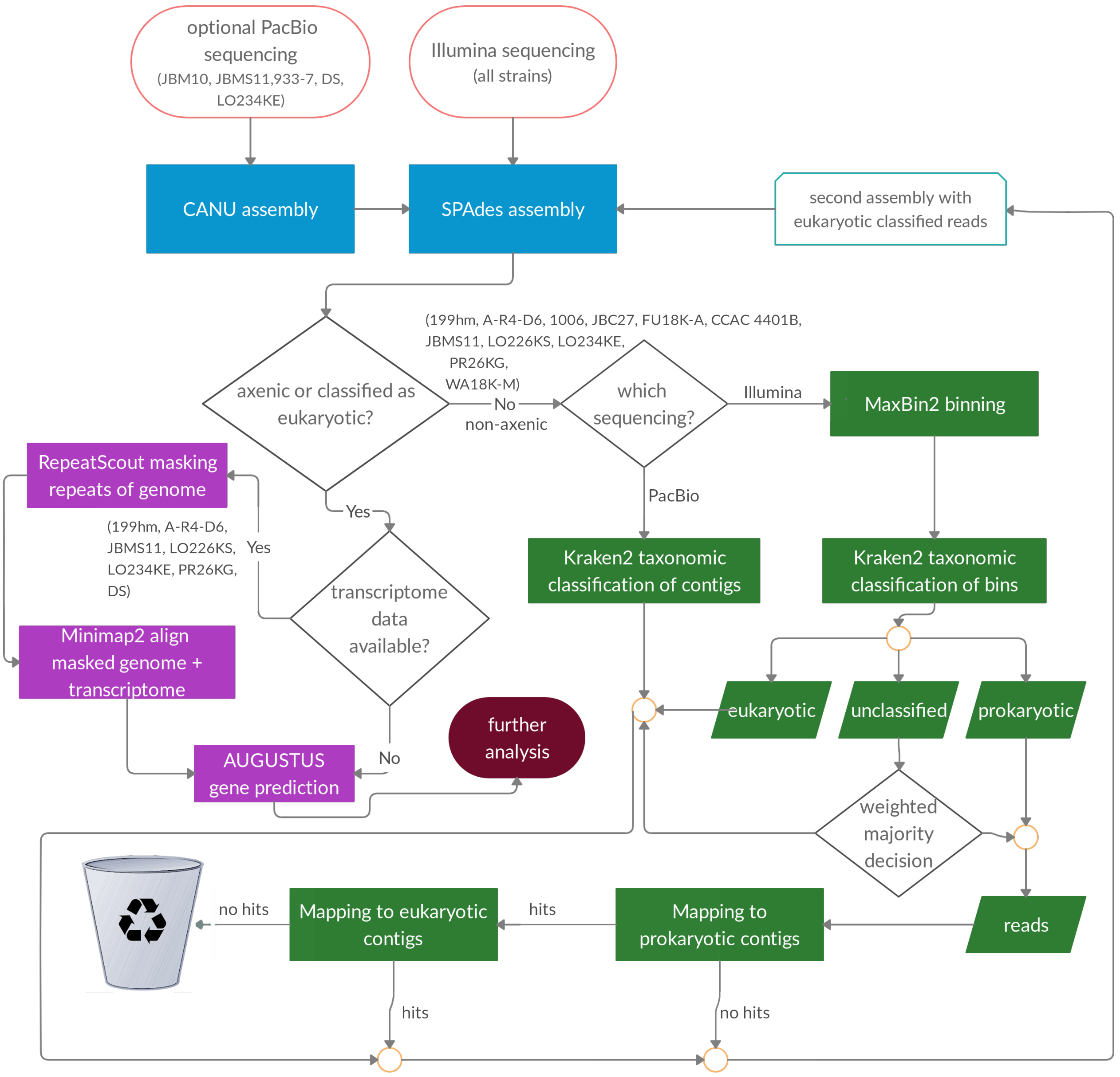

Figure S3: **Proportion of gene assignments for each strain.** Composition of annotated genes according to the second highest hierarchy level of the KEGG functional groups.

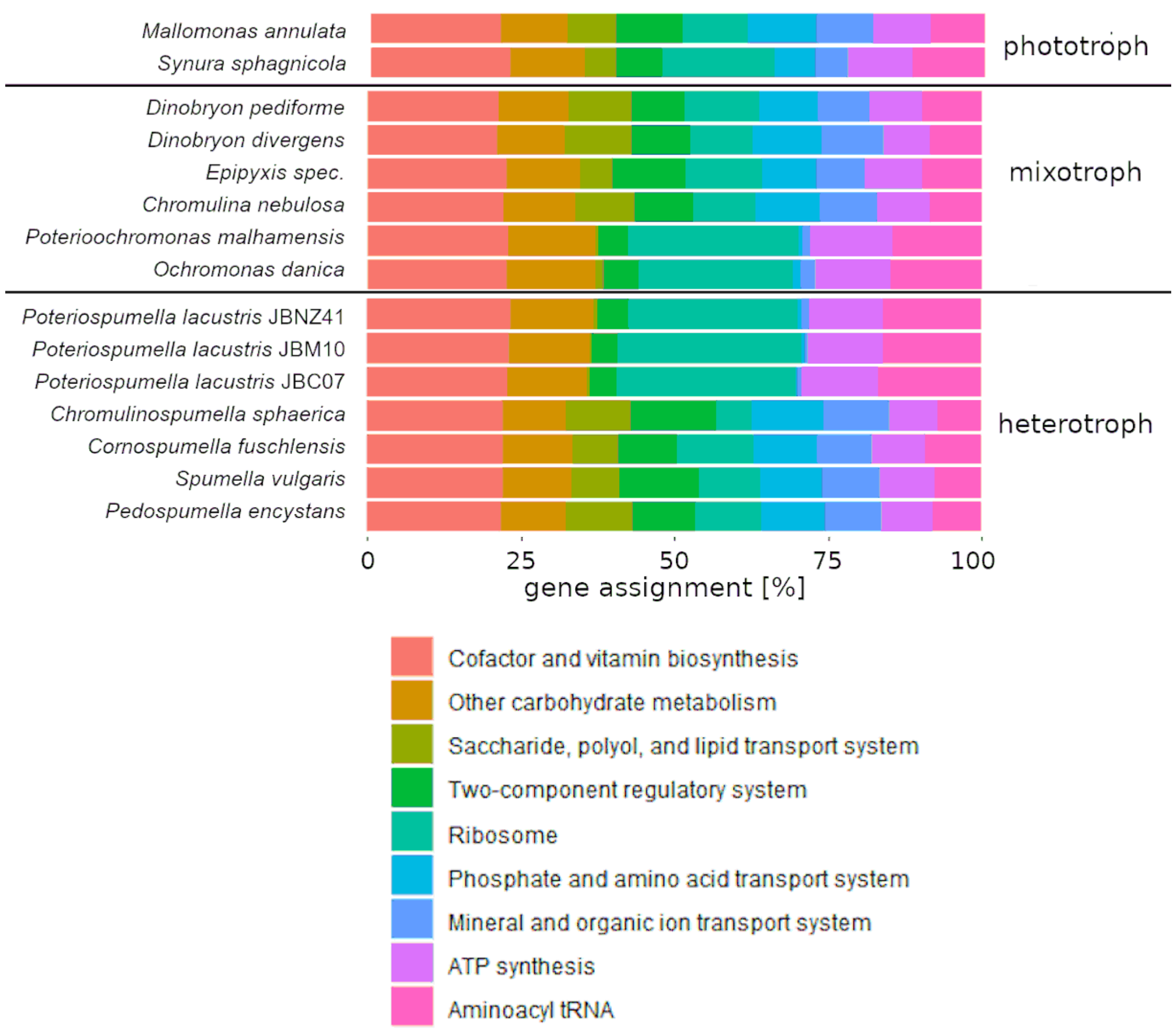

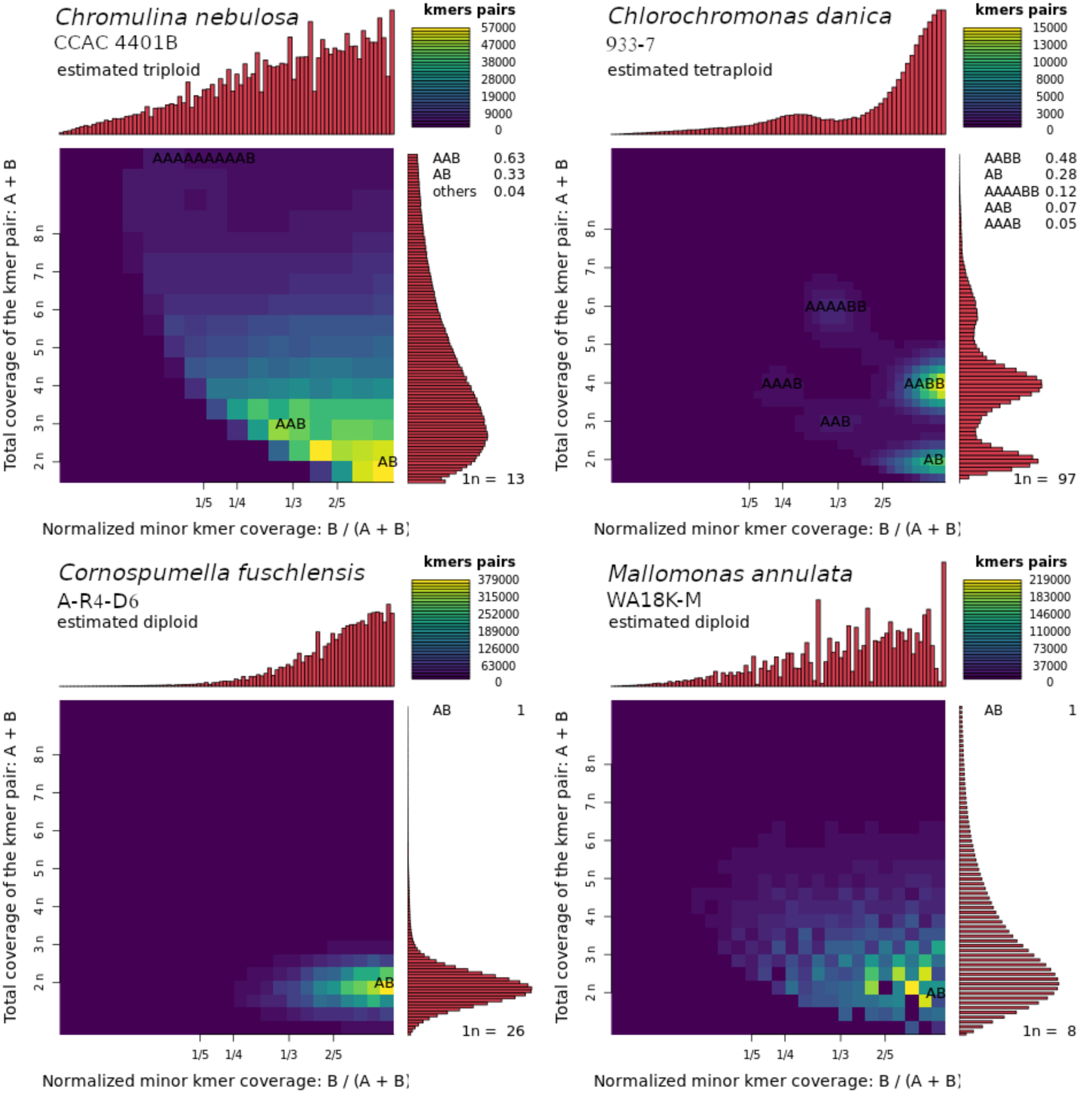

Figure S4: **K-mer based ploidy estimation.** The heatmap reflects the coverage of k-mer pairs differing by one base. The ratio of the characters A to B represents the ratio of these k-mer pairs (e.g., 67% ATGTC and 33% ATGTT conforms AAB). The coverage distribution on the right side indicates ploidy levels (left scale). The distribution on the top side is based on the coverage normalized by the ratio.

n = average k-mer coverage, k-mer size = 21

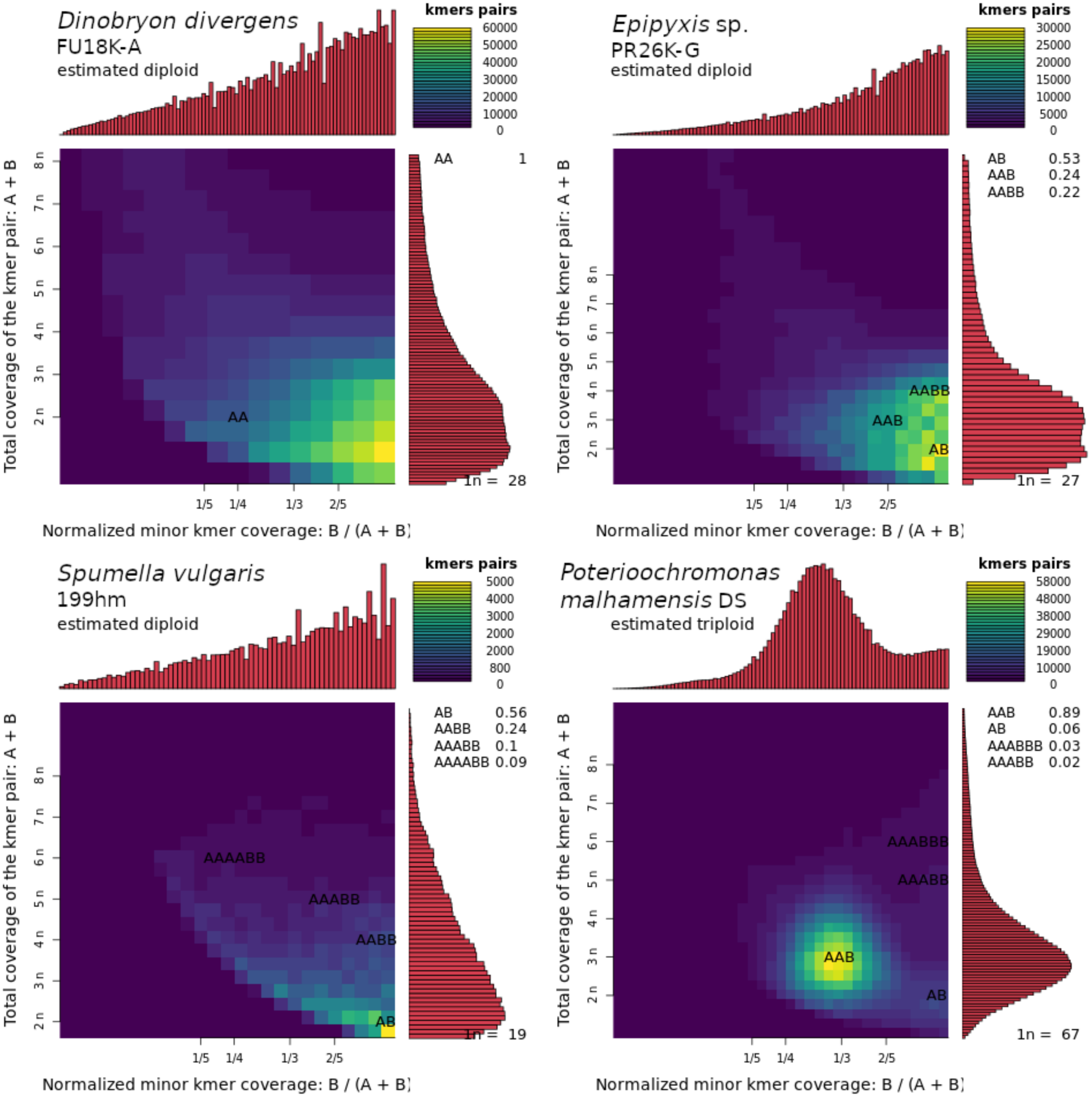

Figure S5: **K-mer based ploidy estimation.** The heatmap reflects the coverage of k-mer pairs differing by one base. The ratio of the characters A to B represents the ratio of these k-mer pairs (e.g., 67% ATGTC and 33% ATGTT conforms AAB). The coverage distribution on the right side indicates ploidy levels (left scale). The distribution on the top side is based on the coverage normalized by the ratio.
n = average k-mer coverage, k-mer size = 21

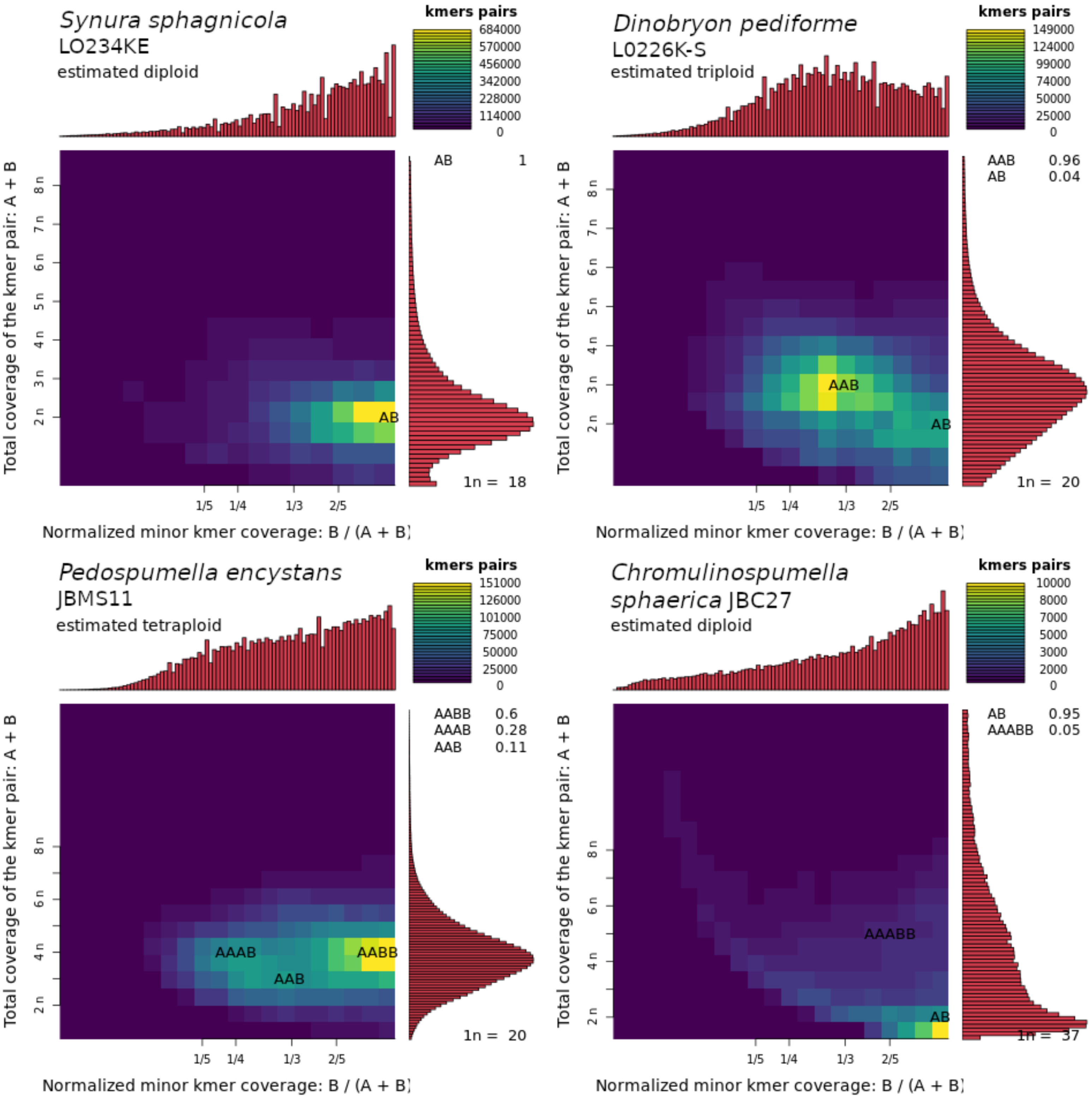

Figure S6: **K-mer based ploidy estimation.** The heatmap reflects the coverage of k-mer pairs differing by one base. The ratio of the characters A to B represents the ratio of these k-mer pairs (e.g., 67% ATGTC and 33% ATGTT conforms AAB). The coverage distribution on the right side indicates ploidy levels (left scale). The distribution on the top side is based on the coverage normalized by the ratio.

n = average k-mer coverage, k-mer size = 21

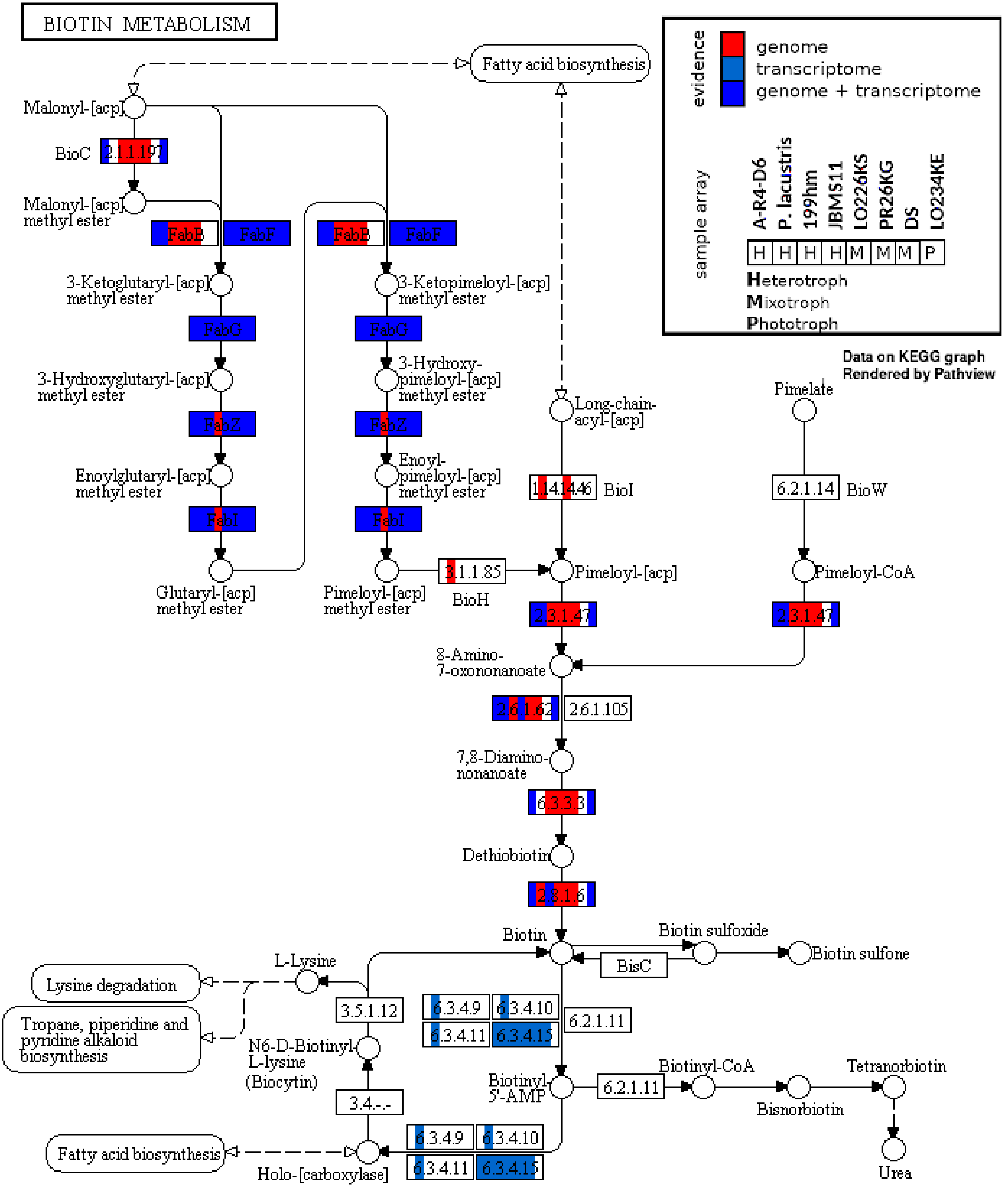

Figure S7: **Biotin metabolism.** Newly identified genes by genome sequencing within this study are marked in red.

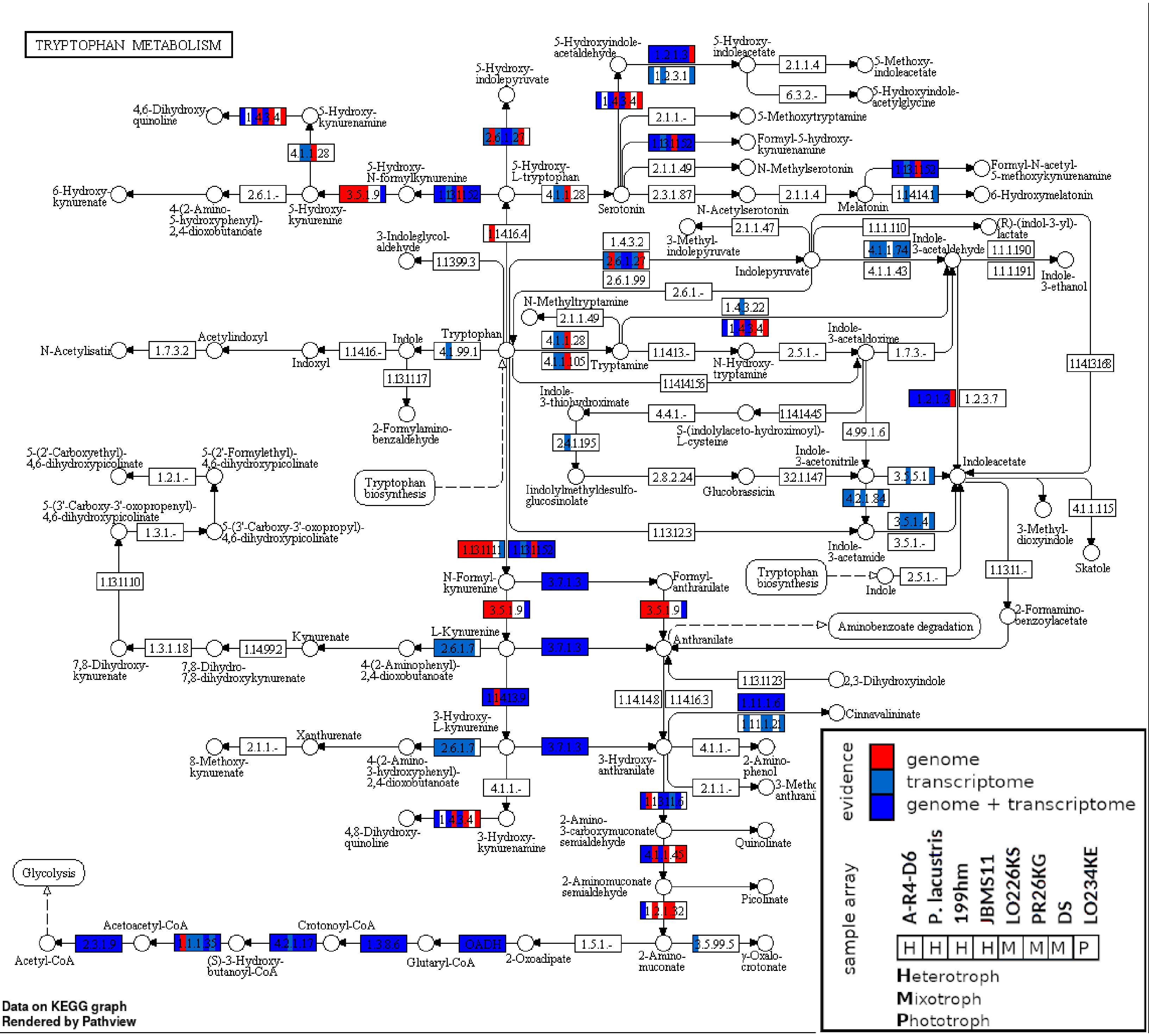

Figure S8: **Tryptophan metabolism.** Newly identified genes by genome sequencing within this study are marked in red.

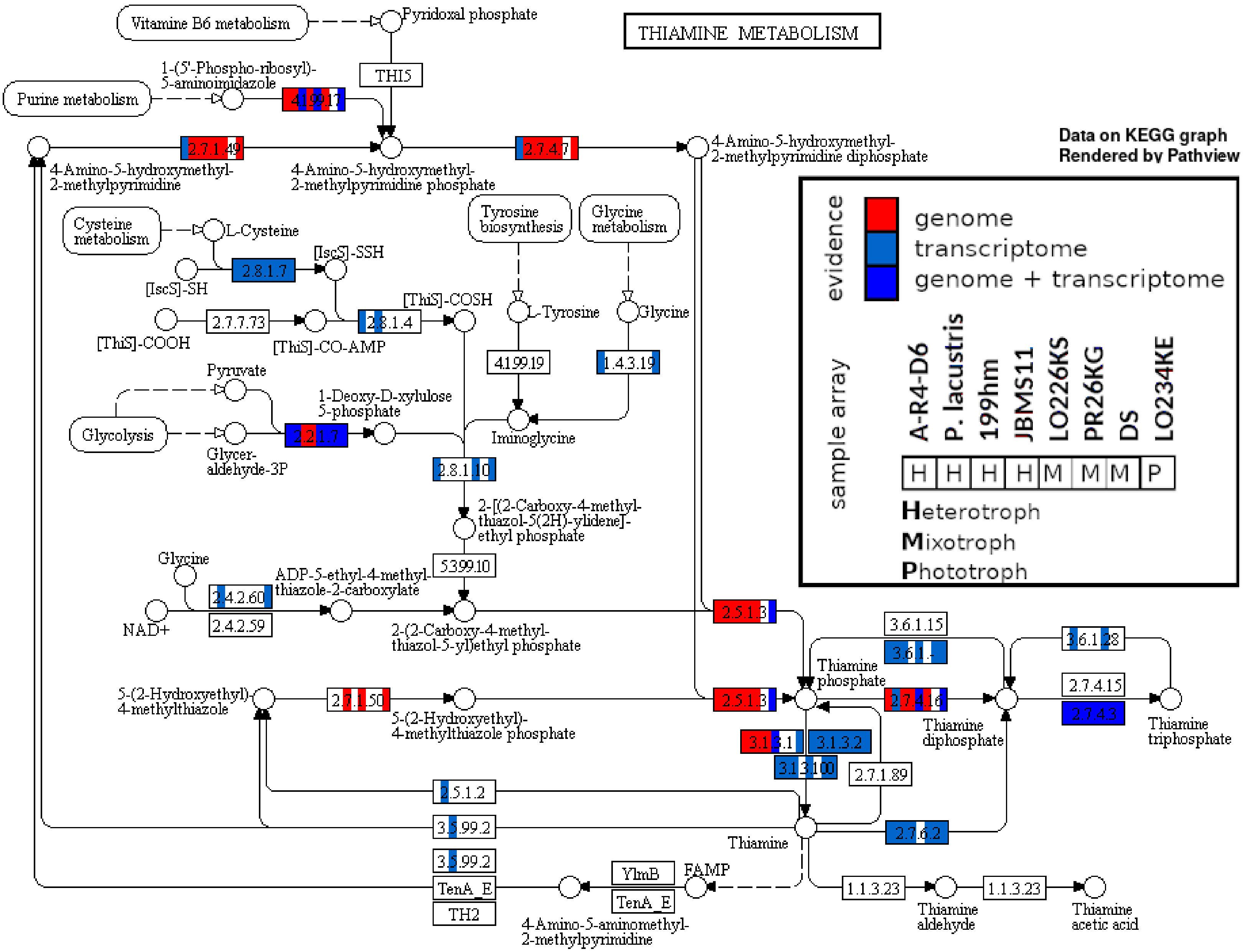

Figure S9: **Thiamine metabolism.** Newly identified genes by genome sequencing within this study are marked in red.

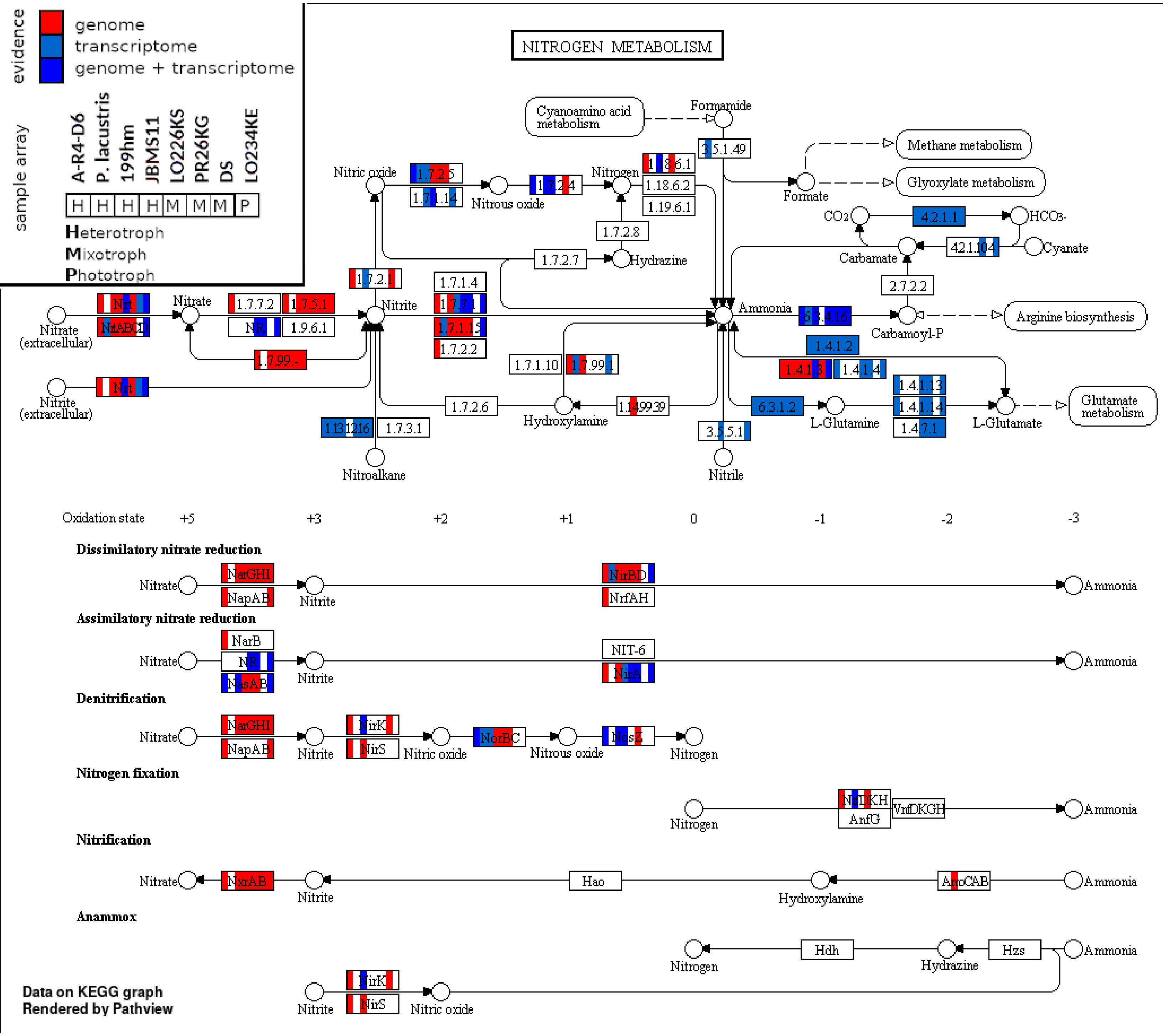

Figure S10: **Nitrogen meatbolism.** Newly identified genes by genome sequencing within this study are marked in red.

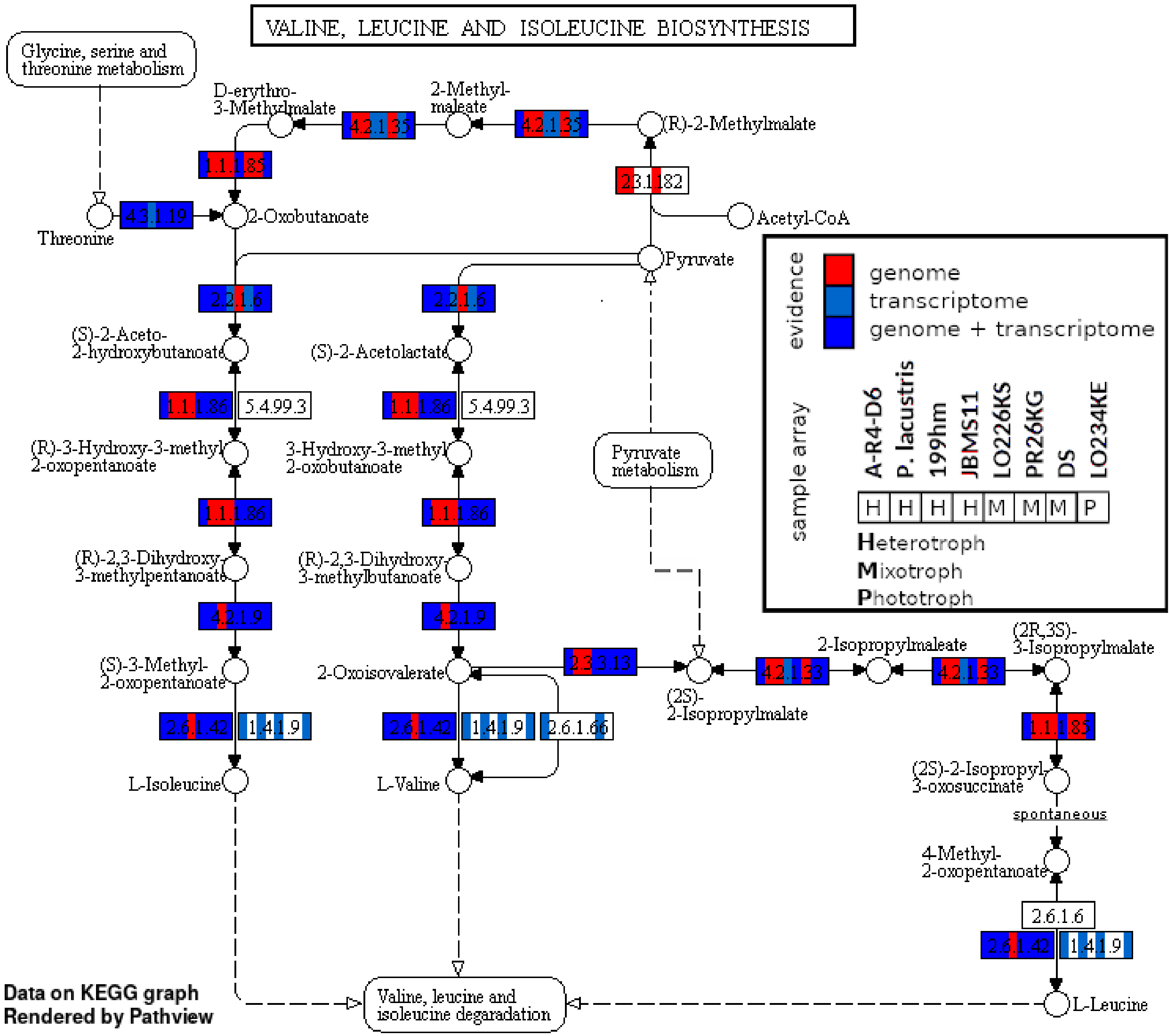

Figure S11: **Valin, leucine and isoleucine biosynthesis.** Newly identified genes by genome sequencing within this study are marked in red.

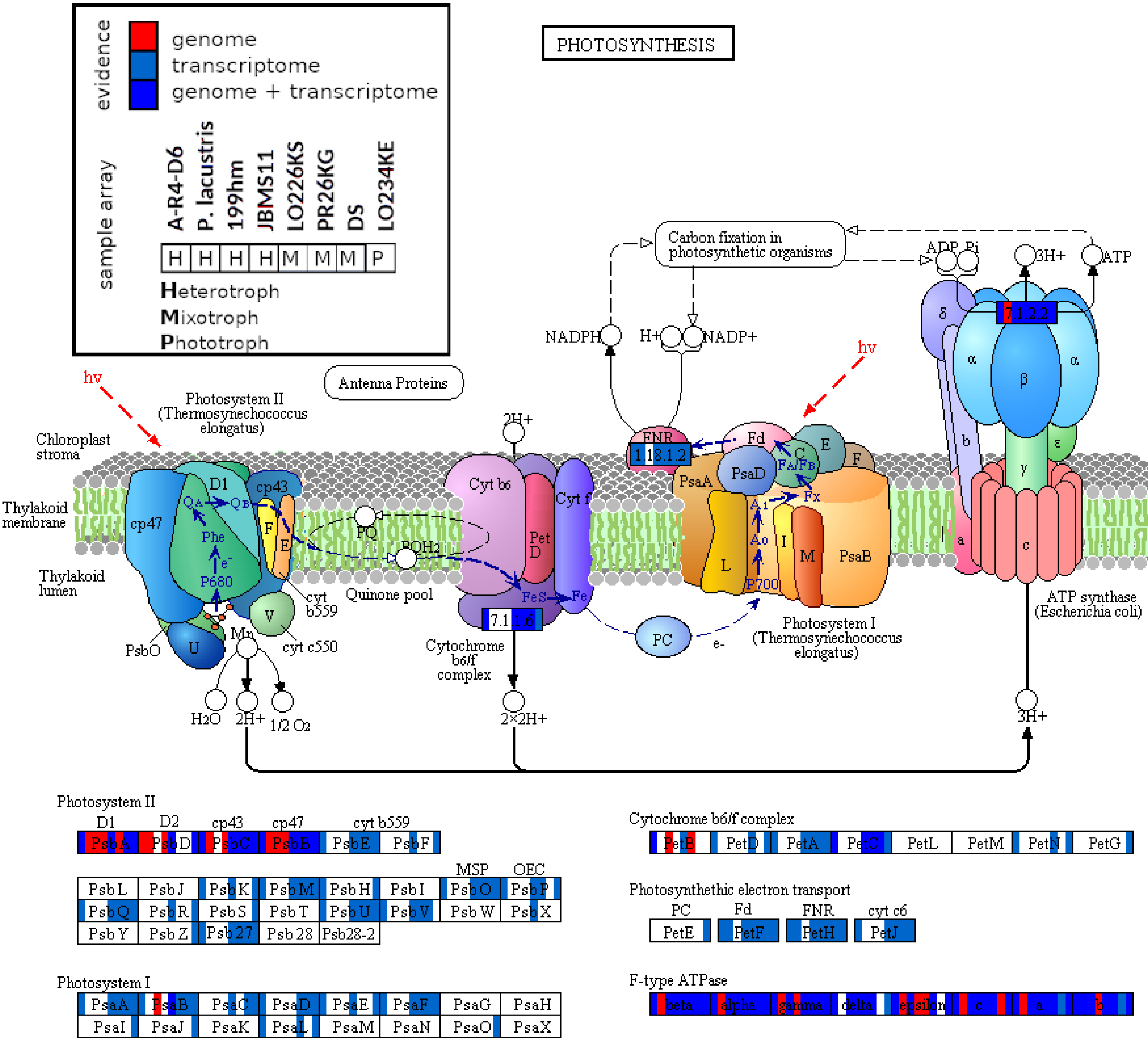

Figure S12: **Photosynthesis.** Newly identified genes by genome sequencing within this study are marked in red.

Figure S13: **Comparison of gene density and genome size by method.** Genome size estimates based on flow cytometry and assembly differ, which in turn effects the gene density, that depends on it. There are the throphic modes **p**hototrophy, **m**ixotrophy and **h**eterotrophy. Error bars represent standard deviations. **a.** Gene density based on genome assembly. **b.** Gene density based on flow cytometry. **c.** The Genome size according to fow cytometry.

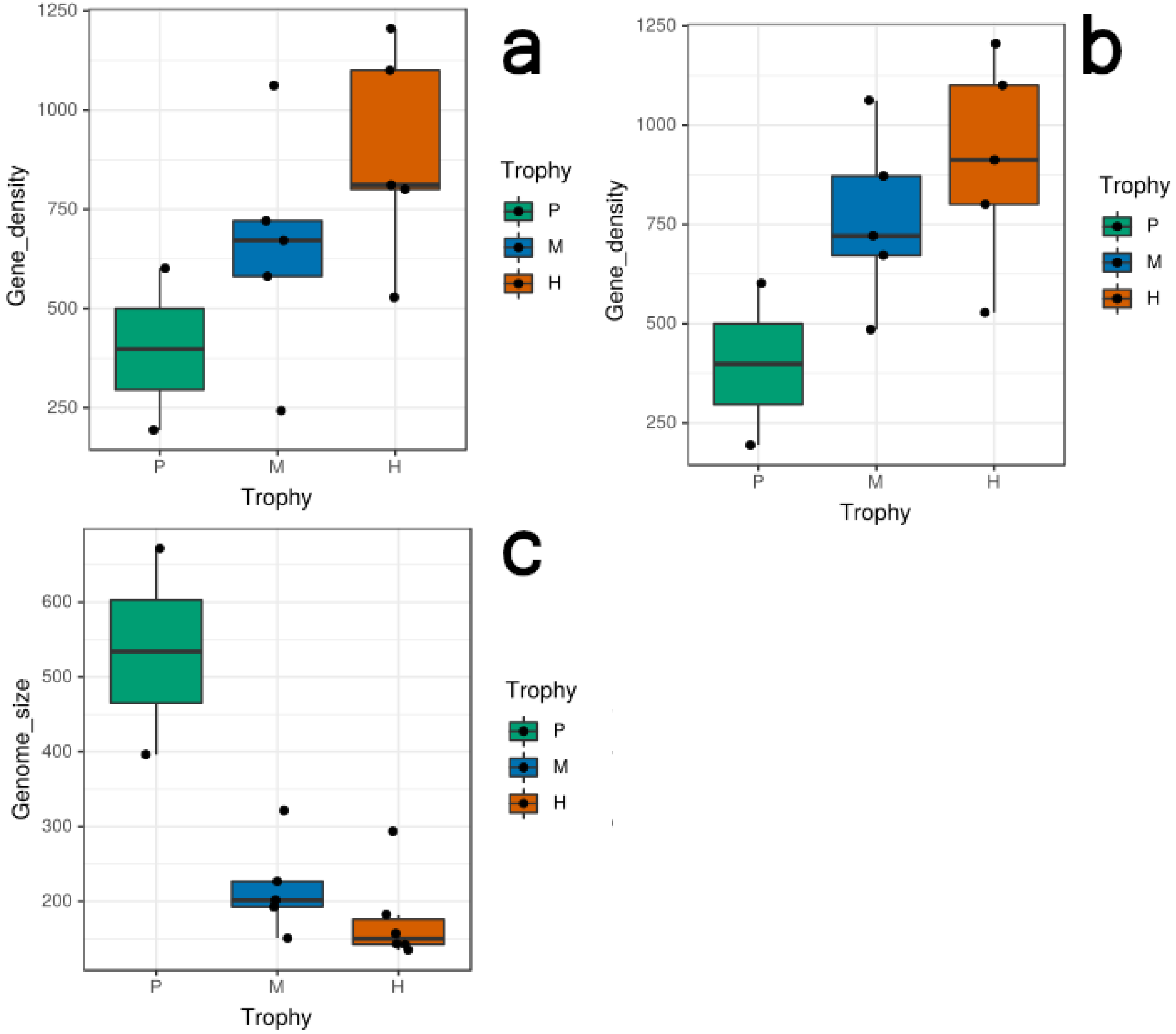

Table S6: Gene counts based on KEGG hierarchy

strain	Unkown	Environmental information processing	Nucleotide and amino acid metabolism	Carbohydrate and lipid metabolism	Genetic information processing	Energy metabolism	Metabolism	Cellular processes	Gene set	Secondary metabolism
1006	34685	2466	2430	1431	1189	582	373	134	132	105
199hm	27572	2102	2009	1195	969	504	298	116	140	101
933-7	18245	374	1341	1100	1594	371	297	158	33	26
AR4D6	25872	1879	2077	1324	1278	539	355	133	98	83
Chromulina	46702	2387	2431	1467	1204	575	390	128	108	102
DS	16556	223	1138	962	1484	353	241	169	18	14
Epipyxis	13011	962	1123	682	589	271	195	53	85	47
FU18K-A	54285	2435	2320	1388	1198	571	361	141	106	95
JBC27	23840	2457	2055	1105	471	481	293	45	155	94
JBMS11	24504	1336	1573	1017	1122	401	290	121	59	53
LO226KS	30057	2160	2446	1543	1514	612	428	157	111	96
LO234KE	27122	1114	1934	1392	1845	535	388	226	73	60
WA18K-M	29562	1634	1700	1081	908	450	281	90	73	75
JBC07	26915	310	2039	1799	3093	626	511	383	13	31
JBM10	25591	291	1965	1732	2970	579	475	360	16	29
JBNZ41	29296	473	2297	1966	3155	669	556	394	33	38
KEGG	122875	4982	5846	3859	4135	1489	1074	509	265	214
photo_union	47750	2231	2765	1881	2070	763	507	253	117	101
photo_intersection	8934	517	869	592	683	222	162	63	29	34
mixo_union	90463	4155	4438	2847	2736	1112	778	318	202	176
mixo_intersection	2561	50	278	209	343	81	58	32	5	4
hetero_union	83503	4373	5360	3591	3979	1378	994	489	237	197
hetero_intersection	1084	13	127	100	102	35	27	18		1
('photo', 'mixo')_union	97196	4369	4781	3078	3064	1216	839	369	214	183
('photo', 'mixo')_intersection	2078	38	237	175	281	70	50	25	5	3
('photo', 'hetero')_union	100219	4676	5609	3740	4058	1435	1029	498	254	207
('photo', 'hetero')_intersection	911	11	105	87	84	30	24	14		
('mixo', 'hetero')_union	122106	4981	5845	3857	4132	1487	1073	508	265	214

Gene counts based on KEGG hierarchy

('mixo', 'hetero')_intersection	744	4	88	64	65	24	20	9		1
('photo', 'mixo', 'hetero')_union	122875	4982	5846	3859	4135	1489	1074	509	265	214
('photo', 'mixo', 'hetero')_intersection	691	4	79	62	59	23	18	8		
