# Supplementary material for: Nutrient-driven genome evolution revealed by comparative genomics of chrysomonad flagellates
Source: Commun Biol. 2021 Mar 12;4:328. doi: 10.1038/s42003-021-01781-3 (PMC7954800; doi:10.1038/s42003-021-01781-3)
Supplement: Supplementary file 3 — Description of Additional Supplementary Files [file 42003_2021_1781_MOESM3_ESM.pdf]

Description of Additional Supplementary Files

File name: Supplementary Data 1

Description: The Supplementary Data 1 lists the occurrence of genes in transcriptomic vs genomic data as shown in figure 2.
