# Supplementary material for: Nutrient-driven genome evolution revealed by comparative genomics of chrysomonad flagellates
Source: Commun Biol. 2021 Mar 12;4:328. doi: 10.1038/s42003-021-01781-3 (PMC7954800; doi:10.1038/s42003-021-01781-3)
Supplement: Supplementary file 5 — Reporting Summary [file 42003_2021_1781_MOESM5_ESM.pdf]

nature research

Corresponding author(s): Stephan Majda
Last updated by author(s): Oct 5, 2020

# Reporting Summary

Nature Research wishes to improve the reproducibility of the work that we publish. This form provides structure for consistency and transparency in reporting. For further information on Nature Research policies, see our Editorial Policies and the Editorial Policy Checklist.

## Statistics

For all statistical analyses, confirm that the following items are present in the figure legend, table legend, main text, or Methods section.

| n/a | Confirmed | |
|---|---|---|
| ☐ | ☒ | The exact sample size (*n*) for each experimental group/condition, given as a discrete number and unit of measurement |
| ☒ | ☐ | A statement on whether measurements were taken from distinct samples or whether the same sample was measured repeatedly |
| ☐ | ☒ | The statistical test(s) used AND whether they are one- or two-sided<br>*Only common tests should be described solely by name; describe more complex techniques in the Methods section.* |
| ☒ | ☐ | A description of all covariates tested |
| ☐ | ☒ | A description of any assumptions or corrections, such as tests of normality and adjustment for multiple comparisons |
| ☐ | ☒ | A full description of the statistical parameters including central tendency (e.g. means) or other basic estimates (e.g. regression coefficient) AND variation (e.g. standard deviation) or associated estimates of uncertainty (e.g. confidence intervals) |
| ☐ | ☒ | For null hypothesis testing, the test statistic (e.g. *F*, *t*, *r*) with confidence intervals, effect sizes, degrees of freedom and *P* value noted<br>*Give P values as exact values whenever suitable.* |
| ☒ | ☐ | For Bayesian analysis, information on the choice of priors and Markov chain Monte Carlo settings |
| ☒ | ☐ | For hierarchical and complex designs, identification of the appropriate level for tests and full reporting of outcomes |
| ☒ | ☐ | Estimates of effect sizes (e.g. Cohen's *d*, Pearson's *r*), indicating how they were calculated |

*Our web collection on statistics for biologists contains articles on many of the points above.*

## Software and code

Policy information about availability of computer code

| | |
|---|---|
| Data collection | NCBI (Release 2018-11-01), KEGG (Release 2014-06-23) and UniProt database (Release 2017-09-18), were used for taxonomic classification |
| Data analysis | We used a custom pipline combining: SPAdes (v3.13.0), CANU (v1.8), MaxBin2 (v2.2.5), Kraken2 (v2.0.7 60), MetaBAT(v2.12.1),Bowtie2 (v.2.3.0), BUSCO software (v3.0.1), smudgeplot (v0.1.3), AUGUSTUS (v3.3), RepeatScout (v1.0.5), BEDTools (v2.28), Diamond (v0.9.10.111), OrthoFinder (v2.2.6), Pathview (v1.24.0), W-IQ-TREE (v1.6.11) and Minimap2 (v.2.9-r720)<br>details see methods section |

For manuscripts utilizing custom algorithms or software that are central to the research but not yet described in published literature, software must be made available to editors and reviewers. We strongly encourage code deposition in a community repository (e.g. GitHub). See the Nature Research guidelines for submitting code & software for further information.

## Data

Policy information about availability of data

All manuscripts must include a data availability statement. This statement should provide the following information, where applicable:

- Accession codes, unique identifiers, or web links for publicly available datasets
- A list of figures that have associated raw data
- A description of any restrictions on data availability

DNA-sequencing data that support the ndings of this study are available from NCBI BioProject IDs:
PRJNA546545 and PRJNA548251.

# Field-specific reporting

Please select the one below that is the best fit for your research. If you are not sure, read the appropriate sections before making your selection.

☐ Life sciences ☐ Behavioural & social sciences ☒ Ecological, evolutionary & environmental sciences

For a reference copy of the document with all sections, see nature.com/documents/nr-reporting-summary-flat.pdf

# Ecological, evolutionary & environmental sciences study design

All studies must disclose on these points even when the disclosure is negative.

| | |
|---|---|
| Study description | The study compare genome characteristics, pan/core genome and gene annotation of 16 chrysophyte strains. We track down the genomic fingerprints of the eco-evolutionary constraints linked to the varying significance of nutrient and carbon shortage. |
| Research sample | chrysophyceae, culture (5 axenic, 11 non-axenic) |
| Sampling strategy | n/a |
| Data collection | n/a |
| Timing and spatial scale | n/a |
| Data exclusions | no data was excluded |
| Reproducibility | All data analysis steps are repeatable. |
| Randomization | n/a |
| Blinding | n/a<br>Blinding has no effect on genome analysis. |

Did the study involve field work? ☐ Yes ☒ No

# Reporting for specific materials, systems and methods

We require information from authors about some types of materials, experimental systems and methods used in many studies. Here, indicate whether each material, system or method listed is relevant to your study. If you are not sure if a list item applies to your research, read the appropriate section before selecting a response.

## Materials & experimental systems

| n/a | Involved in the study | |
|---|---|---|
| ☒ | ☐ | Antibodies |
| ☐ | ☒ | Eukaryotic cell lines |
| ☒ | ☐ | Palaeontology and archaeology |
| ☒ | ☐ | Animals and other organisms |
| ☒ | ☐ | Human research participants |
| ☒ | ☐ | Clinical data |
| ☒ | ☐ | Dual use research of concern |

## Methods

| n/a | Involved in the study | |
|---|---|---|
| ☒ | ☐ | ChIP-seq |
| ☒ | ☐ | Flow cytometry |
| ☒ | ☐ | MRI-based neuroimaging |

## Eukaryotic cell lines

Policy information about cell lines

| | |
|---|---|
| Cell line source(s) | cultures were from culture collection University Duisburg-Essen |
| Authentication | See Table 5: General information of examined species for references |
| Mycoplasma contamination | Cell lines were not tested to mycoplasma contamination. |
| Commonly misidentified lines<br>(See ICLAC register) | n/a |
